# RAD sequencing and genomic simulations resolve hybrid origins within North American *Canis*

**DOI:** 10.1098/rsbl.2015.0303

**Published:** 2015-07

**Authors:** L. Y. Rutledge, S. Devillard, J. Q. Boone, P. A. Hohenlohe, B. N. White

**Affiliations:** 1 Biology Department, Trent University, 2140 East Bank Drive, Peterborough, Ontario, K9J 7B8 Canada; 2 Université de Lyon, F-69000, Lyon; Université Lyon 1; CNRS, UMR5558, Laboratoire de Biométrie et Biologie Evolutive, F-69622, Villeurbanne, France; 3 Floragenex Inc., Eugene, OR 97405, USA; 4 Department of Biological Sciences, University of Idaho, Moscow, ID 83844, USA

**Keywords:** conservation genomics, eastern wolf, evolution, hybridization

## Abstract

Top predators are disappearing worldwide, significantly changing ecosystems that depend on top-down regulation. Conflict with humans remains the primary roadblock for large carnivore conservation, but for the eastern wolf (*Canis lycaon*), disagreement over its evolutionary origins presents a significant barrier to conservation in Canada and has impeded protection for grey wolves (*Canis lupus*) in the USA. Here, we use 127 235 single-nucleotide polymorphisms (SNPs) identified from restriction-site associated DNA sequencing (RAD-seq) of wolves and coyotes, in combination with genomic simulations, to test hypotheses of hybrid origins of *Canis* types in eastern North America. A principal components analysis revealed no evidence to support eastern wolves, or any other *Canis* type, as the product of grey wolf × western coyote hybridization. In contrast, simulations that included eastern wolves as a distinct taxon clarified the hybrid origins of Great Lakes-boreal wolves and eastern coyotes. Our results support the eastern wolf as a distinct genomic cluster in North America and help resolve hybrid origins of Great Lakes wolves and eastern coyotes. The data provide timely information that will shed new light on the debate over wolf conservation in eastern North America.

## 1. Introduction

Carnivores are disappearing at an alarming rate, threatening top-down regulation, ecosystem resiliency and biodiversity worldwide [1]. With their need for expansive spaces and sufficient prey, large predators have an ecological life history that directly conflicts with human interests. This incompatibility has caused a long-standing history of widespread persecution that brought top predators like wolves to the brink of extinction in Europe [2] and North America [3]. The twenty-first century, however, has witnessed some recovery of large carnivores in Europe [4] and the USA [1], suggesting restoration is possible.

Few species present more of a challenge to conservation than those within the *Canis* genus. Eastern wolves (*Canis lycaon*) from Algonquin Provincial Park, Ontario [5] faced targeted extermination during the first half of the twentieth century [6], and high mortality owing to hunting and trapping outside park boundaries during the second half [7]. Since the turn of the millennium, however, the biggest threat to their long-term persistence has been disagreement over their evolutionary history. Although genomics holds promise for improving conservation efforts [8], varying interpretation of genome-wide single-nucleotide polymorphism (SNP) data from wolves and coyotes [9,10] has, so far, only added to the confusion among non-geneticists and policy makers, to the detriment of eastern wolf conservation in Canada and grey wolf (*Canis lupus*) conservation in the USA.

The status of the eastern wolf is currently being re-assessed by the Committee on the Status of Endangered Wildlife in Canada; its assessment has been delayed and hinges largely on the genetic distinction of wolves in Algonquin Park. Similarly, the delisting of grey wolves from the US Endangered Species List has been controversial [11–13], but most recently has been criticized for relying on false assumptions about mutually exclusive historical distributions of eastern and grey wolves in 22 of the eastern states [14,15]. Many factors have contributed to the painstakingly long assessment process in both countries, but part of the conflict has been inadvertently fuelled by the decision of the United States Fish and Wildlife Service to formally recognize (then not recognize, then re-recognize) the eastern wolf as a species, distinct from the grey wolf. Clearly, resolving the evolutionary origins of eastern wolves from Algonquin Park and grey wolves from the Great Lakes states is a key factor in moving forward with wolf conservation in eastern North America. Doing so is particularly opportune for ecosystems where biodiversity is threatened by excessive herbivory from a pandemic overabundance of white-tailed deer (*Odocoileus virginianus*) [16], and because current funding levels for biodiversity conservation are insufficient under species/site-specific terrestrial frameworks [17].

There are two prevailing evolutionary models for North American *Canis*: (i) a two-species model that identifies grey wolves (*C. lupus*) and (western) coyotes (*Canis latrans*) as distinct species that gave rise to various hybrids, including the Great Lakes-boreal wolf (also known as Great Lakes wolf), the eastern coyote (also known as Coywolf/brush wolf/tweed wolf), the red wolf and the eastern wolf [9]; and (ii) a three-species model that identifies the grey wolf, western coyote and eastern wolf (*C. lycaon*) as distinct species, where Great Lakes-boreal wolves are the product of grey wolf × eastern wolf hybridization, eastern coyotes are the result of eastern wolf × western coyote hybridization, and red wolves are considered historically the same species as the eastern wolf, although their contemporary genetic signature has diverged owing to a bottleneck associated with captive breeding [10]. A main criticism of the three-species model is the small number of autosomal genetic markers used to differentiate eastern wolves [9]. Alternatively, data supporting the two-species model may be subject to ascertainment bias associated with SNP genotyping based on the domestic dog genome and insufficient sampling of representative eastern wolves [10]. To test the hypothesis that eastern wolves arose from grey wolf × western coyote hybridization, we used RADSeq [18] of samples that are representative of the various *Canis* types (electronic supplementary material, table S1) to produce genotypes at 127 235 (127K) SNPs based on a grey wolf genomic assembly. We then simulated hybrid profiles of different generations that allowed us to compare observed and expected genotypes under various hybridization scenarios, thus elucidating the potential for hybrid origins of *Canis* types in eastern North America. We used alder v1.03 to infer admixture [19] and TreeMix to calculate *f3* statistics [20] (see supplementary materials for detailed methods). To clarify, we did not consider Great Lakes-boreal wolves to be eastern wolves as some suggest [21]; rather, we used wolves that occur in Algonquin Provincial Park [22] as the best current representation of eastern wolves.

## 2. Results and discussion

We obtained high-quality RADSeq data with a median depth of coverage between 27 and 93× (electronic supplementary material, table S2). There were 197 263 putative RAD loci in the final filtered set, which represents 17.8 Mbp of putative low copy grey wolf genomic reference sequence. After filtering for bi-allelic SNPs (see electronic supplementary material), genotypes at 127 235 SNP loci for each of 17 individuals of five different *Canis* types (electronic supplementary material, table S2) were generated for further analysis.

**Figure 1.**
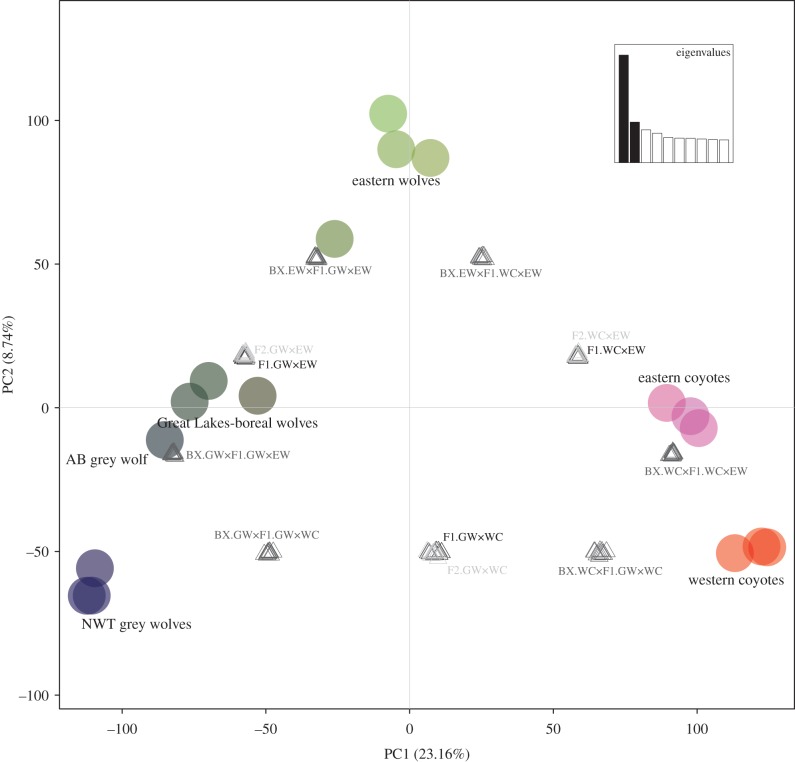
Colour plot of principal components analysis of genome-wide SNP data with simulated hybrid genomes. Analysis of 127 235 SNPs on *Canis* samples referenced to a grey wolf genome assembly. Coloured dots represent observed data and triangles represent simulated hybrid data. Grey wolves are from Northwest Territories and Alberta, Great Lakes-boreal wolves are from northern Ontario, eastern wolves are from Algonquin Provincial Park, eastern coyotes are from southern Ontario, and western coyotes are from Saskatchewan. F1.GWxWC = first-generation grey wolf × western coyote hybrids; F2.GWxWC = second-generation grey wolf × western coyote hybrids; BX.GWxF1.GWxWC = backcross of F1.GWxWC with grey wolves; BX.WCxF1.GWxWC = backcross of F1.GWxWC with western coyotes. F1.GWxEW = first-generation grey wolf × eastern wolf hybrids; F2.GWxEW = second-generation grey wolf × eastern wolf hybrids; BX.GWxF1.GWxEW = backcross of F1.GWxEW with grey wolves; BX.EWxF1.GWxEW = backcross of F1.GWxEW with eastern wolves. F1.WCxEW = first-generation western coyote × eastern wolf hybrids; F2.WCxEW = second-generation western coyote × eastern wolf hybrids; BX.EWxF1WCxEW = backcross of F1.WCxEW with eastern wolves; BX.WCxF1.WCxEW = backcross of F1.WCxEW with western coyotes.

Following the genetic clusters species concept [23], a principal components analysis of SNP genotypes was consistent with the existence of a distinct eastern wolf species (figure 1). Simulated grey wolf × western coyote hybrid genomes failed to overlap with any other *Canis* types when projected on the factorial map, but simulated grey wolf × eastern wolf genomes overlapped with observed data for Great Lakes-boreal wolves and simulated eastern wolf × western coyote genotypes overlapped with observed data for eastern coyotes (figure 1). These patterns are consistent with previous suggestions that the eastern wolf is a conduit of gene flow between grey wolves and coyotes [10,21,24], but are in contrast to previous work that analysed 48K SNPs (based on the dog genome) and concluded (under the assumption of the two-species model) that the eastern wolf from Algonquin Park was a grey wolf × western coyote hybrid [9]. Our work, however, differs from that presented in reference [9] in that our dataset is (i) unfettered by ascertainment bias or assumptions of a two-species model (see electronic supplementary material), (ii) uses 80K more loci and (iii) implements a sampling and analytical design intended to specifically test hypotheses of hybrid origins for the different *Canis* types.

Tests of admixture were contradictory: alder did not identify eastern wolves as admixed between grey wolves × western coyotes, but was limited by sample size (see electronic supplementary material). The *f3* results suggested various wolf × coyote admixture in both eastern wolves and Great Lakes-boreal wolves, with increasing standard error and decreasing significance as the number of SNP re-sampling blocks increased (see electronic supplementary material). The *f3* analysis failed, however, to detect admixture in eastern coyotes—a cluster for which wolf × coyote ancestry is well documented [24,25]. Further, the *f3* results contradict known behavioural [26] and probable biological [27] reproductive barriers between grey wolves and western coyotes. We suspect that the broad estimates of divergence between grey wolves and western coyotes (approx. 1 Ma) and between eastern wolves and western coyotes (approx. 300 000 Ma), in combination with the contemporary admixture observed in the eastern North American populations, may be impacting the ability of these tests to accurately estimate ancestry. We therefore recommend cautious interpretation of both alder and *f3* results presented here, and suggest more extensive sampling may provide more robust results with sophisticated analytical software (see the electronic supplementary material).

Based on 63 diagnostic SNPs, previous research identified eastern coyotes as a mix of western coyotes, western wolves, eastern wolves and domestic dogs [21]. However, the sample of ‘eastern wolves' used in reference [21] originated primarily from the Great Lakes states (*n* = 14) and only included three samples from Ontario. That sample of eastern wolves, therefore, represents Great Lakes-boreal wolves as a parental reference population rather than what are currently considered representative eastern wolves [22]. If one considers (i) the differences in terminology, and (ii) the lack of representative eastern wolf samples from Algonquin Park in previous genomic studies, our results are consistent with SNP results from other researchers who identified the eastern coyote as having wolf × coyote hybrid ancestry [21], and who suggest Great Lakes wolves are admixed [9]. The differences arise based on the interpretation and representation of the eastern wolf, which in our data is represented by animals from Algonquin Park. These differences in terminology and geographical sampling have hindered conservation of eastern wolves in Canada and grey wolves in the USA. We suggest that the most parsimonious explanation of all genetic data to date, including that of mitochondrial DNA [28], Y-chromosome [24] and genome-wide SNP data (this manuscript and [10]), support eastern wolves from Algonquin Park as a distinctive remnant entity of a historical wolf that most likely occurred across the eastern United States.

## 3. Conclusion

Our findings represent important information for implementing effective endangered species policy in North America. We demonstrate support for the eastern wolf centralized in Algonquin Provincial Park as a distinct genomic cluster, thus providing support for the three-species model of *Canis* evolution. Additionally, our data support previous work indicating wolves in the Great Lakes states as originating from grey wolf × eastern wolf origins [29]. Although we were unable to test alternative evolutionary scenarios (e.g. hybridization followed by drift) with this dataset, and specific admixture tests were inconclusive and contradictory, future work with species-specific SNPs and broader sampling may allow a more comprehensive comparison of the alternatives. The recognition of the eastern wolf as a separate species does not exclude the possibility that a grey wolf × eastern wolf hybrid animal (previously identified as *Canis lupus lycaon,* boreal/Ontario-type [30]), similar to a Great Lakes-boreal wolf currently located in the Great Lakes states and across Manitoba, northern Ontario, and northern Quebec, historically inhabited the northeastern United States alongside eastern wolves, and there is some evidence to support the historical presence of both *Canis* types [10]. The recognition of *C. lycaon* should not, therefore, influence grey wolf delisting decisions in the USA. In the light of the current funding gap for biodiversity conservation [17], and the increased biodiversity, reduced disease and control of invasive species that occurs with top-down regulation [31], wolf conservation could provide a fundamental, cost-effective approach to reduce herbivory, conserve ecosystems and improve biodiversity in the troubled landscapes of eastern North America.

## Supplementary Material

SupplementaryMaterialsBiolLettJune1_2015.docx

## Supplementary Material

SupplementaryFileBiolLettF3ResultsJune1_2015.xlsx

## Data accessibility

Wolf/Coyote DNA Bank at Trent University.
