# Correction to ‘RAD sequencing and genomic simulations resolve hybrid origins within North American *Canis*’

**DOI:** 10.1098/rsbl.2017.0594

**Published:** 2017-11-01

**Authors:** L. Y. Rutledge, S. Devillard, J. Q. Boone, P. A. Hohenlohe, B. N. White

*Biol. Lett.* **11**, 20150303. (Published online 8 July 2015) (doi:10.1098/rsbl.2015.0303)

The data associated with Rutledge *et al*. [1] are now available on the Dryad Digital Repository at http://dx.doi.org/10.5061/dryad.pr318 [2].
