# Supplementary material for: RAD sequencing and genomic simulations resolve hybrid origins within North American Canis
Source: Biol Lett. 2015 Jul;11(7):20150303. doi: 10.1098/rsbl.2015.0303 (PMC4528444; doi:10.1098/rsbl.2015.0303)
Supplement: SupplementaryMaterialsBiolLettJune1_2015.docx [file rsbl20150303supp1.docx]

Supplementary Materials:

| **Table S1.** Samples used for RAD-seq experiment. Coordinates are measured and accurate to 100m except for Saskatchewan and Northwest Territories (NWT) where specific locations are not known and estimates are centralized but varied so they do not overlap when mapping. The AB Gray Wolf is from Jasper National Park and co-ordinates are estimated near the town of Jasper. NRDPFC = Natural Resources DNA Profiling and Forensic Centre; APP=Algonquin Provincial Park. | | | | |
| --- | --- | --- | --- | --- |
| **NRDPFC Sample ID** | ***Canis* Type** | **Location** | **Latitude** | **Longitude** |
| CAN001024 | Gray Wolf | Northwest Territories | 62.518022 | -114.366302 |
| CAN001051 | Gray Wolf | Northwest Territories | 62.446324 | -114.498138 |
| CAN001055 | Gray Wolf | Northwest Territories | 62.415816 | -114.436340 |
| DB179893 | Gray Wolf | Alberta | 52.867472 | -117.978058 |
| CAN002356 | Western Coyote | Saskatchewan | 50.733848 | -109.048920 |
| CAN002360 | Western Coyote | Saskatchewan | 50.864044 | -106.824188 |
| CAN002369 | Western Coyote | Saskatchewan | 50.863178 | -104.959259 |
| CAN004255 | Eastern Wolf | Ontario - APP | 45.496133 | -78.429533 |
| CAN004534 | Eastern Wolf | Ontario - APP | 45.580231 | -78.390554 |
| DB179508 | Eastern Wolf | Ontario - APP | 45.99335 | -78.26558 |
| DB179509 | Eastern Wolf | Ontario - APP | 45.33048 | -78.95967 |
| CAN004210 | Eastern Coyote | Ontario - Southern | 44.200463 | -81.149188 |
| CAN004216 | Eastern Coyote | Ontario - Southern | 44.061691 | -80.653732 |
| CAN004232 | Eastern Coyote | Ontario - Southern | 44.108311 | -80.556987 |
| CAN004172 | Great Lakes-Boreal Wolf | Ontario - Northern | 49.087152 | -91.534080 |
| CAN004186 | Great Lakes-Boreal Wolf | Ontario - Northern | 49.766775 | -86.460085 |
| CAN004243 | Great Lakes-Boreal Wolf | Ontario - Northern | 49.217126 | -80.723843 |

| **Table S2.** RAD-seq quality summary. | | | |
| --- | --- | --- | --- |
| **Sample ID** | ***Canis* type** | **Observed Reads/Sample** | **Median Sequencing Depth** |
| CAN004210 | Eastern Coyote | 25,645,436 | 64x |
| CAN004216 | Eastern Coyote | 26,990,843 | 68x |
| CAN004232 | Eastern Coyote | 20,031,125 | 53x |
| CAN004255 | Eastern Wolf | 31,224,328 | 77x |
| DB179508 | Eastern Wolf | 37,183,968 | 80x |
| DB179509 | Eastern Wolf | 23,750,915 | 60x |
| CAN004534 | Eastern Wolf | 21,680,044 | 57x |
| CAN004172 | Great Lakes-Boreal Wolf | 32,345,788 | 62x |
| CAN004186 | Great Lakes-Boreal Wolf | 20,537,795 | 51x |
| CAN004243 | Great Lakes-Boreal Wolf | 13,157,245 | 35x |
| CAN001024 | Gray Wolf | 20,436,315 | 42x |
| CAN001051 | Gray Wolf | 30,616,772 | 55x |
| CAN001055 | Gray Wolf | 14,892,486 | 34x |
| DB179893 | Gray Wolf | 8,846,424 | 27x |
| CAN002356 | Western Coyote | 45,700,149 | 93x |
| CAN002360 | Western Coyote | 36,421,679 | 79x |
| CAN002369 | Western Coyote | 10,814,803 | 32x |

**Supplementary Methods and Results:**

*Sample Selection*

To avoid sampling admixed individuals that are known to occur in Ontario, even in Algonquin Park [1], we selected samples based on their geographic sampling location in combination with their prior assignment (with probability Q≥0.8) to each *Canis* group, according to Bayesian clustering analysis of 12 autosomal microsatellites in the program STRUCTURE [2] with parameterization as follows: F-model for correlated allele frequencies with 5 initial runs at 500,000 iterations after a burn-in of 250,000 with K varying from 1 - 8. We inferred the most-likely number of clusters using methods described [3]. Subsequent runs were similarly conducted 10 times at optimal K, and Q scores were averaged across runs. In addition, Eastern Wolf samples from Algonquin Provincial Park were collected in three different years (2002, 2006, 2010), had three different mtDNA haplotypes (C14, C17, C19) and were unrelated (r<0) based on relatedness estimators [4, 5] calculated on 12 autosomal microsatellites in GenAlEx 6.501 [6].

*Sample Preparation*

Seventeen *Canis* tissue samples from across Canada were retrieved from the Natural Resources DNA Profiling and Forensic Centre (NRDPFC; www.nrdpfc.ca) Wolf and Coyote DNA Bank at Trent University (wolf.nrdpfc.ca; table S1). Total genomic DNA was extracted with a DNeasy Blood and Tissue kit (Qiagen) according to manufactuer’s directions but with the following changes: DNA was eluted in 400 μL of TrisEDTA (TE) buffer solution (to retrieve maximal DNA) and the concentrated down to 25 μL with a Amicon 30K Centrifugal filter (Millipore) followed by the addition of 25 μL TE buffer. Following fluorometric quantification with Quant-iT PicoGreen reagents (Life Technologies), concentration standardization, and purity assessment on an agarose gel and a Nanodrop 8000 (Thermo-Fisher), 3µg of each sample was submitted to Floragenex (Portland, Oregon; www.floragenex.com), a genomic services company specializing in RAD-sequencing.

*Library construction and sequencing*

Floragenex used the RAD-Seq protocol described by [7,8,9]. Following restriction digestion with *SbfI*, individual sequence identifier ‘barcodes’ and sequence adapters were ligated to genomic DNA. We used a single-end 1x100bp approach to sequence pooled samples outwards from cut sites on an Illumina HiSeq2000 (Illumina Inc.). Samples were de-multiplexed and barcodes trimmed to produce 90 bp fragments.

*RAD-seq data processing, mapping, and variant detection*

Although the dog genome is complete, we did not wish to bias detection of variation in some unpredictable manner through use of genomic resources acquired from a domestic species. We, therefore, constructed a *de novo* “RAD reference” or “Unitag set” based on a Gray Wolf sample (sample ID CAN001051). We selected the Gray Wolf sample for which preliminary analysis suggested the greatest number of unique RAD tag clusters (330,338) were sequenced (Table S2). We used the program BOWTIE [10], which is optimized for short reads and incorporates sequence quality information, to align reads into clusters (minimum depth of 5x and maximum depth of 500x). We allowed no more than three mismatches among reads within a cluster and a maximum of two haplotypes per Unitag set. Such criteria are intended to filter repetitive or paralogous DNA regions from the RAD references. For heterozygous positions, one base was selected randomly as the reference allele.

We used BOWTIE to map reads for each individual to the Gray Wolf RAD reference genome, again including sequence quality information and setting a maximum of three mismatches. No more than one reference region per read was permitted. The SAMTOOLS pileup module (available at http://github.com/samtools) was used to detect SNPs and call genotypes. Initial filters for SNP calling required a minimum phred score of 15, a minimum of 10X sequence coverage, and a maximum of 15% missing data across samples. For the final dataset, we eliminated SNPs with three or more alleles. Overall, we implemented bioinformatics protocols that provided sufficient coverage to minimize false positive/false negative homozygote detection, and helped avoid mapping to multigene families and repetitive or paralogous regions. SNPs were tabulated and formatted in variant call format (VCF) [11]. We used vcftools (available at http://vcftools.sourceforge.net) and custom scripts to filter VCF files and convert them to the format required by the *adegenet* package [12] for subsequent analyses.

*Summary of Gray Wolf reference clustering and filtering procedure.*

First, 30,616,772 Illumina sequence reads from Gray Wolf sample CAN001051 were trimmed from the 5’ end to a total length of 90 bp. Floragenex used custom perl scripts to cluster 100% identical sequences. Based on the observed coverage distribution, these sequences were posited to be sequence reads from low-copy or single-copy RAD loci in the Gray Wolf genome. Subsequently, a total of 24,009,836 RAD sequence reads were coalesced from the total pool of reads from Gray Wolf sample CAN001051. This represented approximately 78% of the total sequence data from sample CAN001051, which were then ordered into an initial assembly.

We used BWA [13] to condense the Gray Wolf assembly to fasta format and align it to itself. We set the BWA alignment conditions to allow a maximum of two haplotypes per cluster and a maximum of a 3bp mismatch between reference and query sequences (96.6% sequence identify). This self-alignment was used to identify sequences within the assembly that carried substantial homology to one another. Clusters with more than two observed haplotypes were discarded as a potential paralog or duplicated sequence in the assembly.

*Hybrid Genome Simulations & Mapping*

We simulated hybrids of four generations (F1, F2, backcross into both parental populations) for three putative parental population pairs: hybridization GW x WC between Gray Wolves (including NWT and AB, n=4) and Western Coyotes (n=3), GW x EW between Gray Wolves (n=4) and Eastern Wolves (n=4), and, EW x WC between Eastern Wolves (n=4) and Western Coyotes (n=3). First we calculated the mean allele frequencies at each SNP locus in each observed and *a priori* defined parental population. Second we simulated parental populations of n=10 genotypes by sampling allele at each SNP locus within the mean observed allelic frequencies matrix using multinomial distribution. Third, from these parental populations, we associated randomly sampled haploid gametes of each parental population to simulate hybridization and built an F1 population of 10 genotypes. Introgression (F2, backcross into both parental populations) was then simulated following the same rules from the F1 simulated population by randomly associated two F1 gametes (F2), one F1 gamete and one gamete from each simulated parental population (backcross). Simulations used 127,235 SNP loci after filtering for bi-allelic loci (from 197,263 reduced to 127,435) and removing 200 additional loci for which at least one parental population showed no data. Missing data at one SNP locus were replaced by sampling based on the corresponding mean allele frequency in observed SNP data.

Our hybrid genotype simulations are performed within the classical “hybridization” framework used in conservation biology: members of two *a priori* “parental” populations mate and reproduce together to lead to fertile F1 individuals likely introgressing parental genomes. Our hybrid genotype simulations thus reflect current hybridization at a temporal scale of only a few generations.

*Principal Components Analysis*

We performed a principal component analysis (PCA) on allelic frequencies with 127,235 SNP genotypes for 17 individuals. We then projected on the PCA factorial map the simulated hybrid genotypes to assess whether or not some observed SNP genotypes matched with the simulated hybrid SNP genotypes. The basic idea behind this approach is to assess whether some of the observed genotypes, excluding those considered as part of the parental populations in hybrid simulations (GW, WC, EW), are consistent with the genotypes that would be observed if current hybridization occurs between those parental populations. All simulations and analyses were performed in the *adegenet* [12] and *ade4* [14] packages of the *R* software [15]. We implemented the colorplot function in *adegenet* so that colors of observed genotypes result from the summary of individual scores at the three first PCs of the PCA that were translated each into a channel of color (red, green, and blue) in the RGB color system.

*Two- vs Three- Species Model of Evolution*

We also highlight the impact that the *a priori* assumption of a two-species model of evolution can have on the interpretation of the results. We contrasted the results of two PCAs, one performed using only the Gray Wolves and Western Coyotes observed SNP genotypes, i.e. PCA under the two-species model hypothesis, and, the other performed using Eastern Wolves genotypes in addition of those of Gray Wolves and Western Coyotes, i.e. PCA under the three-species model hypothesis. For each of those PCA, all other genotypes (including remaining observed ones as well as simulated ones) were then projected on the factorial map. PCA was first performed under the two-species hypothesis model (Fig. S1) using a sub-sample of the observed SNP genotypes containing only those of the Gray Wolves and Western Coyotes. Then all other genotypes (including remaining observed ones as well as simulated ones) were projected on the factorial map.

Using the two-species model as a working hypothesis, all observed SNP genotypes (GLBW, EW and EC) are projected within the two species GW and WC along the first PC on the factorial map (Fig. S1). Such a two-species hypothesis leads thus to interpret all observed SNP genotypes and simulated SNP genotypes as intermediate of those of GW and WC. This pattern is thus consistent with the idea that GLBW, EW and EC are various hybrids of GW and WC.

On the contrary, performing the PCA under the three-species hypothesis model reveals the importance of two principal components to explain the genotypic variability of the SNP genotypes for the 17 samples (eigenvalues plot, Fig. S2). The second PC clearly differentiated GW and WC at the bottom of the factorial map and EW at the top of the factorial map. Projected SNP genotypes, both observed ones of GLBW and EC and simulated ones, reproduced the overall pattern described in main text based on the PCA performed with all observed genotypes.

Overall, the comparison of both PCAs under either the two- or the three-species model strengthened our results. Indeed, the second PC of the PCA performed on all the observed SNP genotypes was not clearly differentiated from the following ones (eigenvalues plot, Fig. 1, main text). Retaining too many PCs might add random noise, namely over-interpretation, in the description of the pattern of genetic variability [16]. However, the PCA performed under the three-species hypothesis allowed us to rule out the risk of over-interpretation by highlighting two important and meaningful PCs. Finally, performing the PCA without any hypothesis (eigenvalues plot, Fig. 1) did not increase the number of reliable PCs anymore, suggesting that two PCs is the most likely number of PCs to retain.

*Admixture Analysis*

We used ALDER [17] and *f3* statistics [18,19] to test for evidence of admixture among these taxa. ALDER depends on linkage disequilibrium among neighboring loci and haplotype structure to estimate timing of admixture, and variance around estimates of *f3* depends on the non-independence of physically linked loci, so we first placed our RAD loci on a physical map. We aligned the consensus sequences of our GW-derived RAD loci (see above) against the dog reference genome assembly [20]. We used Bowtie2 [21] in end-to-end alignment mode, allowing one mismatch in a seed length of 20bp. This produced unique alignments for 85.9 percent of all RAD loci, so that 121,408 of our previously identified SNPs could be placed on the physical map of the dog genome. We then used this position information for both ALDER and *f3* analyses. ALDER found no evidence for admixture from GW and WC into EW, although jackknife significance testing failed in most cases, presumably because of low samples size of individuals. We were unable to test for admixture in any of the other populations for the same reason.

We also used the dog reference-aligned SNPs to estimate *f3* in a three-population analysis. The *f3* statistic is used to identify correlations in allele frequencies that are not compatible with evolutionary history of groups given an assumed bifurcating tree; *f3* statistics are used to identify admixture in the history of the tested populations [18, 22]. We implemented the three-population test (*f3* statistic) in Treemix [19] for all possible triplets, taking physical linkage into account. We tested significance of *f3* with bootstrapping, grouping SNPs in blocks of 1, 10, 100, or 1000 to span a wide range of possible non-independence of linked loci. Results from the *f3* test are available in the separate Supplementary Excel File. We do, however, have concerns about the interpretation of these results. These concerns are listed below:

1. The *f3* statistic was developed to determine ancient admixture events within human populations. Thus, the test looks for fine scale levels of admixture within a species, rather than testing for significant differentiation among species. We know that Eastern Wolves have been influenced by some level of contemporary admixture with both Great Lakes-Boreal Wolves and Eastern Coyotes [1], and therefore we would expect detection of fine scale influence from Gray Wolves and Western Coyotes within the population of Eastern Wolves (via the Great Lakes-Boreal Wolf and Eastern Coyote populations that are in geographic proximity to the Eastern Wolf population in Algonquin Park). It should not be overlooked that despite low levels of admixture, the Eastern Wolf population in Algonquin Park has remained distinct from the surrounding *Canis* types since the 1960s when the first research sampling was done [23]. Although the mechanism is not well understood, there is some evidence from the Y-chromosome that assortative mating may be involved [1].
2. The *f3* results are contradicted by several lines of genetic and non-genetic evidence.
   1. In addition to the PCA and simulations presented here, the *f3* results conflict with evidence of a 3-species model of *Canis* evolution suggested by both mtDNA [24] and the Y-chromosome [25]. Both maternal and paternal markers indicate a historical branching of 3 *Canis* types. Extensive hybridization/introgression, however, has blurred these lineages within the context of the contemporary geographic landscape.
   2. The *f3* results contradict what we know ecologically and biologically about these species. Ecologically, it is well documented and generally agreed upon that western Gray Wolves and Western Coyotes do not interbreed in the wild [26] and are in fact antagonistic to each other [27]. Biologically, there is some suggestion of genetic incompatibility between western Gray Wolves and Western Coyotes as demonstrated by the difficulty of artificial insemination resulting in pregnancy and/or live births [28].
3. It is well documented that the Eastern Coyote is the product of a hybridization event between a Wolf (either Eastern Wolf or Great Lakes-Boreal Wolf) and a Western Coyote [29, 30]. Coyotes arrived in eastern North America about 100 years ago and as they inhabited new landscapes they interbred with dwindling Eastern Wolves [31] and/or Great Lakes-Boreal Wolves [32]. The *f3* results failed to identify admixture in this cluster, which brings into question the ability of the analysis to accurately detect admixture and clarify genomic ancestry in this dataset.
4. Although ALDER did not detect Gray Wolf x Western Coyote admixture in Eastern Wolves, estimates of linkage disequilibrium require large numbers of individuals. Our dataset is clearly limited by the small sample size (despite the large number of SNPs) in this analysis as demonstrated by the inability of the software to fit decay curves for LD and the failure in some cases in the jackknife resampling.

In consideration of all the lines of evidence, we feel it would be inappropriate to interpret the *f3* results as conclusive while ignoring the extensive genetic, biological, and ecological data that contradict the *f3* statistics. In this regard, we recommend caution in the interpretation of the *f3* results as it could result in inaccurate inferences from genomic data in isolation of other important information. For this reason we focus on the individual-based PCA results, which we believe are more appropriate here where we have a large number of high-confidence genotypes across a few representative individuals from each taxon.

*Limitations of the Data*

Although individual sample sizes are small, the number of markers used is substantially larger than previously reported; the small sample size is somewhat compensated by the high genomic coverage of the dataset. In addition, the samples selected for analysis are representative of the larger geographic clustering pattern based analysis on genome-wide SNP data. Based on clustering analysis of 48K SNP data in the program STRUCTURE conducted in [33], coyotes from southern Ontario cluster with other northeastern US coyote populations; Great Lakes-Boreal Wolves from northern Ontario cluster with those from the Great Lakes states of Wisconsin and Minnesota; western coyotes from the Canadian prairie province of Manitoba cluster with those from western United States; and Gray Wolves from the Northwest Territories cluster with those from Alaska and Yellowstone National Park. So, although more samples would provide more variation for hybrid simulations, it is improbable that further sampling would qualitatively alter the support for the three-species model in both the PCA and hybridization simulations. We suspect that further sampling would only increase the ability of the PCA of all SNP genotypes to more clearly identify two strongly meaningful PCs in the dataset (Fig. S1, S2). As noted in the main text, further sampling may lend itself to tests of alternative evolutionary scenarios (ancient hybridization followed by drift over many generations) and provide more reliable results from sophisticated tests of genomic ancestry.

In cases where simulated hybrid genotypes do not overlap with observed genotypes, conclusions may be that either such hybrid genotypes do not exist in the wild because hybridization leading to such genotypes never occurred or that such genotypes were never sampled.

We acknowledge that the geographic sampling is incomplete and we cannot rule out the possibility that Gray Wolf x Western Coyote hybrids simply were not sampled in our observed data. However, this does not influence the interpretation that the samples included in the dataset, which are representative of the larger geographic distribution, did not arise from Gray Wolf x Western Coyote hybridization. Red wolves are missing from the analysis due to lack of availability of samples at the time of analysis. Our results do not exclude the possibility that hybrid Great Lakes-Boreal Wolves existed historically alongside Eastern Wolves in the eastern United States, and our data do not rule out the possibility that Eastern Wolves underwent selection and/or drift after an ancient hybridization event, although this seems unlikely since present-day gray wolves and coyotes do not interbreed in the wild [26].

**Fig. S1:** Principal component analysis (PCA) of genome-wide SNP data under the two-species hypothesis model. Colored circles and triangles are observed genotypes. See legend for various symbols that represent simulated hybrids genotypes (see Materials and Methods). NWT = Northwest Territories, AB = Alberta, GLBW = Great Lakes-Boreal Wolves, EW = Eastern Wolves, EC = Eastern Coyotes.

**Fig. S2.** Principal component analysis (PCA) of genome-wide SNP data under the three-species hypothesis model. Colored circles and triangles are observed genotypes. See legend for various symbols that represent simulated hybrids genotypes (see Materials and Methods). NWT=Northwest Territories, AB = Alberta, GLBW = Great Lakes-Boreal Wolves, EC = Eastern Coyotes.

List of Abbreviations:

AB: Alberta; APP: Algonquin Provincial Park; BX: backcross; BWA: Burrows-Wheeler Alignment; EC: eastern coyote; EW: eastern wolf; GLBW: Great Lakes-boreal wolf; GW: gray wolf; NRDPFC: Natural Resources DNA Profiling & Forensic Centre; NWT: Northwest Territories; PCA: principal components analysis; RAD-seq: restriction-site associated DNA sequencing; SNP: single nucleotide polymorphism; VCF: variant call format; WC: Western Coyote;

**Supplementary References:**

1. Rutledge LY, Garroway CJ, Loveless KM, Patterson BR. 2010a Genetic differentiation of eastern wolves in Algonquin Park despite bridging gene flow between coyotes and Gray wolves. *Heredity* **105**, 520–531 (doi:10.1038/hdy.2010.6).

2. Falush D, Stephens M, Pritchard JK. 2003 Inference of population structure using multilocus genotype data: linked loci and correlated allele frequencies. *Genetics*. **164**, 1567–1587.

3. Evanno G, Regnau S., Goudet J. 2005 Detecting the number of clusters of individuals using the software STRUCTURE: a simulation study. *Mol. Ecol.* **14**, 2611–2620 (doi:10.1111/j.1365-294X.2005.02553.x).

4. QuellerDC, Goodnight KF. 1989 Estimating relatedness using genetic markers. *Evolution* **43**, 258–275 (doi:10.2307/2409206).

5. Lynch M, Ritland K. 1999 Estimation of pairwise relatedness with molecular markers. *Genetics* **152**, 1753–1766.

6. Peakall R, Smouse PE. 2012 GenAlEx 6.5: genetic analysis in Excel. Population genetic software for teaching and research – an update. *Bioinformatics* **28**, 2537–2539 (doi:10.1093/bioinformatics/bts460).

7. Baird NA, Etter PD, Atwood TS, Currey MC, Shiver AL, Lewis ZA, Selker EU, Cresko WA, Johnson EA. 2008 Rapid SNP discovery and genetic mapping using sequenced RAD markers. *PLoS ONE* **3**, e3376 (doi:10.1371/journal.pone.0003376).

8. Emerson KJ, Merz CR, Catchen JM, Hohenlohe PA, Cresko WA, Bradshaw WE, Holzapfel CM. 2010 Resolving postglacial phylogeography using high-throughput sequencing. *P. Natl. Acad. Sci. USA.* **107**, 16196–16200 (doi:10.1073/pnas.1006538107).

9. Hohenlohe PA, Bassham S, Etter PD, Stiffler N, Johnson EA, Cresko WA. 2010 Population genomics of parallel adaptation in threespine stickleback using sequenced RAD tags. *PLoS Genet.* **6**, e1000862 (doi:10.1371/journal.pgen.10000862).

10. Langmead B, Salzberg S. 2012 Fast gapped-read alignment with Bowtie2. *Nat. Methods.* **9**, 357-359 (doi:10.1038/nmeth.1923).

11. Danecek P, Auton A, Abecasis G, Albers CA, Banks E, DePristo MA, Handsake RE, Lunter G, Marth GT, Sherry ST, McVean G, Durbin R, 1000 Genomes Project Analysis Group. 2011 The variant call format and VCFtools. *Bioinformatics* **27**, 2156–2158 (doi:10.1093/bioinformatics/btr330).

12. Jombart T, Ahmed I. 2011 *adegenet 1.3-1*: new tools for the analysis of genome-wide SNP data. *Bioinformatics*. **27**, 3070-3071 (doi:10.1093/bioinformatics/btr521).

13. Li H, Durbin R. 2009 Fast and accurate short read alignment with Burrows-Wheeler Transform. *Bioinformatics*. **25**, 1754–60 (doi:10.1093/bioinformatics/btp324).

14. Dray S, Dufour AB. 2007 The ade4 package: implementing the duality diagram for ecologists. *J. Stat. Softw*. **22**, 1–20.

15. R Core Team. 2014 R: A language and environment for statistical computing. R Foundation for Statistical Computing, Vienna, Austria; (http://www.R-project.org/).

16. Peres-Neto PR, Jackson D, Somers K. 2005 How many principal components? Stopping rules for determining the number of non-trivial axes revisited. *Comput. Stat. Data. An.* **49**, 974–997 (10.1016/j.csda.2004.06.015).

17. Loh P-R, Lipson M, Patterson N, Moorjani P, Pickrell JK, Reich D, Berger B. Inferring admixture histories of human populations using linkage disequilibrium. *Genetics* **193**, 1233-1254 (doi:10.1534/genetics.112.147330).

18. Reich D, Thangaraj K, Patterson N, Price AL, Singh L. 2009 Reconstructing Indian population history. *Nature* **461**, 489-494 (doi:10.1038/nature08365).

19. Pickrell JK, Pritchard JK. 2012 Inference of population splits and mixtures from genome-wide allele frequency data. *PLOS Genet*. **8**, e1002967 (doi:10.1371/journal.pgen.1002967).

20. Lindblad-Toh K, Wade CM, Mikkelsen TS, Karlsson EK, Jaffe DB, Kamal M, Clamp M, Chang JL, Kulbokas EJ, Zody MC, Mauceli E, *et al.* 2005 Genome sequence, comparative analysis and haplotype structure of the domestic dog. *Nature* **438,** 803-819 (doi:10.1038/nature04338).

21. Langmead B, Trapnell C, Pop M, Salzberg SL. 2009 Ultrafast and memory-efficient alignment of short DNA sequences to the human genome. *Genome Biol.* **10**, R25 (doi:10.1186/gb-2009-10-3-r25).

22. Patterson N, Moorjani P, Luo Y, Mallick S, Rohland N, Zhan Y, Genschoreck T, Webster T, Reich D. 2012 Ancient admixture in human history. *Genetics* **192**, 1065–1093 (doi:10.1534/genetics.112.145037).

23. Rutledge LY, White BN, Row JR, Patterson BR. 2012 Intense harvesting of eastern wolves facilitated hybridization with coyotes. *Ecol. Evol.* **2,** 19-33 (doi:10.1002/ece3.61).

24. Rutledge LY, Patterson BR, White BN. 2010b Analysis of *Canis* mitochondrial DNA demonstrates high concordance between the control region and ATPase genes. *BMC Evol. Biol.* **10,** 215 (doi:10.1186/1471-2148-10-215).

25. Wilson PJ, Rutledge LY, Wheeldon TJ, Patterson BR, White BN. 2012 Y-chromosome evidence supports widespread signatures of three-species *Canis* hybridization in eastern North America. *Ecol. Evol.* **2,** 2325-2332 (doi:10.1002/ece3.301).

26. Mech LD. 2011 Non-genetic data supporting genetic evidence for the Eastern Wolf. *Northeastern Nat*. **18**, 521-526 (doi:http://dx.doi.org/10.1656/045.018.0409).

27. Merkle JA, Stahler DR, Smith DW. 2009 Interference competition between gray wolves and coyotes in Yellowstone National Park. *Can. J. Zool.* **87,** 56-63 (doi:10.1139/Z08-136).

28. Mech LD, Christensen BW, Asa CS, Callahan M, Young JK*.* 2014 Production of hybrids between western gray wolves and western coyotes. *PLoS ONE*, **9**, e88861 (doi:10.1371/journal.pone.0088861).

29. Wilson PJ, Grewal SK, Mallory FF, White BN. 2009 Genetic characterization of hybrid wolves across Ontario. *J. Hered.* **100(suppl 1)**, S80-S89 (doi:10.1093/jhered/esp034).

30. Kays R, Curtis A, Kirchman JJ*.* 2010 Rapid adaptive evolution of northeastern coyotes via hybridization with wolves. *Biol. Lett.* **6**, 89-93 (doi:10.1098/rsbl.2009.0575).

31. Wheeldon T, White BN. 2009 Genetic analysis of historic western Great Lakes region wolf samples reveals early *Canis lupus/lycaon* hybridization. *Biol. Lett.* **5,** 101-104 (doi:10.1098/rsbl.2008.0516).

32. Leonard JA, Wayne RK. 2008 Native Great Lakes wolves were not restored. *Biol. Lett..* **4,** 95-98 (doi:10.1098/rsbl.2007.0354).

33. vonHoldt BM, Pollinger JP, Earl DA, Knowles JC, Boyko AR, Parker H, Geffen E, Pilot M, Jedrzejewski W, Jedrzejewska B, *et al.* 2011 A genome-wide perspective on the evolutionary history of enigmatic wolf-like canids. *Genome Res.* **21**, 1294–1305 (doi:10.1101/gr.116301.110).
